# Necrotizing Otitis Externa: Insights From a Nationwide Database on In‐Hospital Outcomes and Characteristics

**DOI:** 10.1002/oto2.70253

**Published:** 2026-05-20

**Authors:** Noa Berick, Golda Grinblat, Majd Hajouj, Itzhak Braverman, Isaac Shochat

**Affiliations:** ^1^ Department of Otolaryngology, Head and Neck Surgery Hillel Yaffe Medical Center Hadera Israel; ^2^ The Ruth and Bruce Rappaport Faculty of Medicine Technion Haifa Israel

**Keywords:** demographic factors, health disparities, nationwide inpatient sample, necrotizing otitis externa, osteomyelitis

## Abstract

**Objectives:**

To evaluate demographic and hospital‐related factors associated with length of stay (LOS) in patients hospitalized with necrotizing otitis externa (NOE).

**Study Design:**

Retrospective cohort study.

**Setting:**

Nationwide Inpatient Sample (NIS), 2017 to 2019.

**Methods:**

Patients with a primary diagnosis of NOE were identified from the NIS. Linear regression and additional statistical analyses were performed to assess associations between LOS and variables including age, gender, race, socioeconomic status, hospital type, and comorbidities such as diabetes.

**Results:**

A total of 3580 patients with NOE were included. Increased age was significantly associated with longer LOS (*P* < .001). Black patients had longer hospitalizations compared to white patients (6.1 vs 4.4 days, *P* < .001). Women were hospitalized at a younger age than men (54 vs 60 years, *P* < .001), while men had longer LOS (5.3 vs 4.6 days, *P* = .033). Patients treated in teaching hospitals and those without diabetes had longer LOS. Socioeconomic disparities were evident, with Black patients more likely to reside in lower‐income ZIP code areas (*χ*² = 19.4, *df* = 3, *P* < .001). The number of diagnoses did not differ significantly between Black and non‐Black patients (*t* = 0.758, *df* = 697, *P* = .449).

**Conclusion:**

Elderly, male, Black patients and those treated at teaching hospitals tend to have longer hospitalizations for NOE. Women and non‐diabetic patients are admitted at younger ages. These findings identify demographic and hospital‐related factors that are associated with LOS and support the need for additional research to clarify the factors underlying these disparities.

Necrotizing otitis externa (NOE) is a soft tissue infection of the external ear canal (EAC) that spreads from the EAC into the temporal bone and skull base. The infection gradually causes bone destruction, replacing it with granulation tissue.^1^, ^2^ The prevalent causing agent is *Pseudomonas aeruginosa*,^3^ also various bacterial species, such as *Proteus mirabilis*, along with fungal species, have been documented.^3^, ^4^, ^5^, ^6^ For this organism to colonize in the EAC, it requires a moist environment and the absence of effective host defense. Therefore, NOE mainly affects immunocompromised individuals and is mainly known as a Diabetes Mellitus (DM) complication.^1^, ^2^ Early diagnosis and initial aggressive therapy may prevent severe complications and disease spread.^7^, ^8^

NOE is a severe and infrequent disease with sparsity of studies addressing its social and demographic characteristics. Mahdayoun et al^9^ reviewed the English and French publications between 1963 and 2011 and confirmed that, generally, the typical patient at risk is an elderly diabetic male, however, more recent studies describe as well nondiabetic and younger patients.^9^

Epidemiologic study conducted on the nationwide inpatient sample (NIS) database between 2002 and 2013 retrieved 8300 cases of NOE mainly focusing on the difference between age groups (pediatric, adult, and elderly) and found most patients requiring hospitalization were nonelderly. Furthermore, almost half of the adult group did not suffer from DM.^10^

The most recent review in English publications between 2011 and 2019^1^ reviewed 284 cases and found strong male preponderance, with male‐to‐female ratio of 3.3 to 1, with an increased prevalence of DM. Additionally, disease‐specific mortality, persistence, and recurrence rates were observed to be consistent in recent years.

The aim of the current NIS‐based study is to analyze hospitalized NOE patients’ characteristics, outcomes, as well as the demographics, ethnics, and social status.

## Methods

Data for this study were obtained from the 2017‐2019 National Inpatient Sample (NIS) database. The NIS is part of the Healthcare Cost and Utilization Project (HCUP) funded by the Agency for Healthcare Research and Quality. It is the largest all‐payer inpatient care database in the United States, comprising data from approximately 20% of hospital admissions nationwide. To minimize sampling bias, the NIS is stratified by geographic region, urban versus rural location, teaching status, and hospital bed size. This stratification ensures that hospitals within each subgroup have equal probability representation in the selected 20% sample. The data are weighted to accurately represent the entire inpatient population, allowing for comprehensive analysis of healthcare utilization and outcomes across the country.

Using the NIS database, all inpatient hospitalizations between 2017 and 2019 with the International Classification of Disease, 10th Revision (ICD‐10), code for NOE (H60.20) listed as the primary diagnosis at hospital discharge were identified. The ICD‐10 codes H60.21, H60.22, and H60.23 for malignant otitis externa were also included. Demographic data in the form of gender, age, race, and median household income for ZIP Code were collected for analysis from the NIS database. Hospital characteristics such as location and teaching status were also included. Patient outcomes including length of stay (LOS) and in‐hospital mortality, defined as patients dying during hospitalization, were evaluated.

While in‐hospital mortality is a critical outcome, our dataset did not include any deaths during the study period, making it unsuitable as a primary outcome for this analysis. Consequently, we focused on LOS as the main outcome. LOS is a significant measure of hospital resource utilization and patient recovery, reflecting the severity and complexity of NOE cases. By identifying factors that influence LOS, we aim to provide insights into improving patient management and optimizing healthcare resources for NOE treatment.

This study was conducted using publicly available, de‐identified data from the NIS. In accordance with institutional policy and national regulations, studies utilizing de‐identified administrative datasets do not require Institutional Review Board approval. Therefore, review by the Hillel Yaffe Medical Center Institutional Review Board was not required.

The statistical analysis was conducted using the Jamovi statistical software version 2.3 (The Jamovi Project, 2022). A correlation matrix was formed between the LOS and the other study variables: age, gender, race, zip code income, hospital characteristics, as well as presence of DM and other immunocompromised situations. One‐way analysis of variance was also used to compare the means of 2 or more independent groups. A linear regression model was constructed to describe the impact of the different variables on hospitalization length‐of‐stay. A *P*‐value of .05 was considered statistically significant.

## Results

### NOE Trends and Patient's Characteristics

Between the years 2017 and 2019, a total of 3580 cases with NOE as the principal diagnosis were identified from the NIS database. The trends in patient characteristics are displayed in Table 1. The mean age was 57.2 years (±21.2), with a slight male preponderance (55.6%). The mean length of hospitalization was 4.9 days (±5.0), with a median LOS of 4 days (±5.03). Among the different races, the highest proportion of patients with NOE were whites (55.9%), followed by Hispanics (20.7%), Black patients (14.2%), followed by Native Americans (0.7%). In addition, 59.2% of NOE patients suffered from DM.

**Table 1:** Demographic and Clinical Characteristics of Patients With Necrotizing Otitis Externa

n	3580
Mean age (SD)	57.2 (21.2)
Female (%)	1590 (44.4)
Mean length of stay (SD)	4.9 (5.0)
Median length of stay (SD)	5 (5.03)
Race (%)	
White	1955 (55.9)
Black	495 (14.2)
Hispanic	725 (20.7)
Asian	110 (3.1)
Native	25 (0.7)
Other	185 (5.3)
ZIP Code income quartile (%)	
1	1135 (32.3)
2	900 (25.6)
3	855 (24.3)
4	625 (17.8)
Location/teaching status (%)	
Rural	240 (6.7)
Urban nonteaching	655 (18.3)
Urban teaching	2685 (75.0)
Diabetes (%)	2125 (59.2)
HIV (%)	30 (0.8)
Hematopoietic malignancy (%)	80 (2.2)

The primary outcome in the current study was the length of hospitalization. Most of the cases were below 25 days of hospitalization. Among the cases exceeding 25 days of hospitalization, 3 exceptional cases had hospital stays longer than 50 days. All 3 patients were men; 2 were white and 1 was black. Their lengths of stay were 91, 55, and 52 days, respectively.

### Hospital LOS

**Figure 1:**
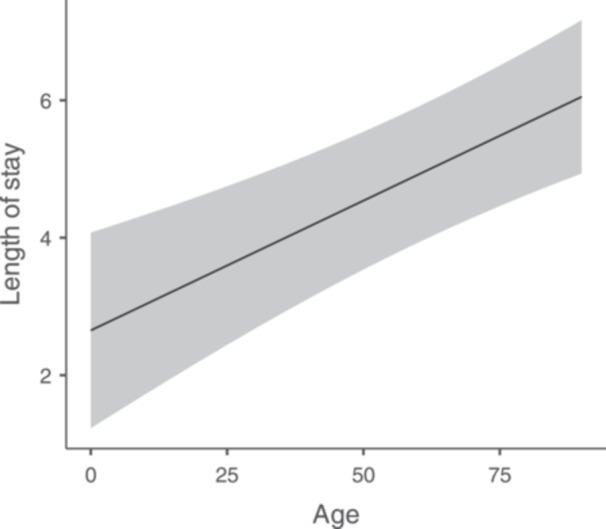
Estimated marginal means of length of stay by age. As age increases, the length of stay also increases in a linear fashion.

In linear regression analysis (Table 2), adjusting for age, gender, race, income, and hospital status—age was found as the most significant predictor of hospitalization length (*P* < .1, *β* = .0378). As age increases, the LOS also increases in a linear fashion (*P* < .001, Figure 1). Race was also found as a significant predictor for the LOS, specifically comparing between blacks and whites (6.1 for blacks vs 4.4 days for whites, *P* < .001, *β* = 1.8485). Interestingly, blacks had a greater variance in the length of hospitalization (5.8 vs 4.2).

**Table 2:** Model Coefficients—Length of Stay

Predictor	Estimate	SE	*t*	*P*
Age	0.0378	0.00873	4.326	<.001
Female‐male	0.5765	0.36592	1.567	.116
Race				
Black‐White	1.8485	0.55257	3.345	<.001
Hispanic‐White	1.0634	0.47514	2.238	.026
Asian‐White	1.2209	1.03977	1.174	.241
Native‐White	0.7236	2.12735	0.340	.734
Other‐White	1.4713	0.81695	1.801	.072
ZIP Code income quartile (%)				
1st‐2nd	−0.4735	0.48289	−0.981	.327
1st‐3rd	−0.8106	0.49454	−1.639	.102
1st‐4th	−0.2500	0.54684	−0.457	.648
Teaching status				
Urban‐Teaching—Rural	−1.2256	0.76971	−1.592	.112
Urban‐Teaching—Urban‐Nonteaching	−1.2654	0.46539	−2.719	.007

Presents the coefficients, standard errors, *t*‐values, and *P*‐values for each predictor variable in the regression model.

Further analyses were conducted to explore potential explanations for the observed results. The relationship between race and ZIP code income levels was examined using a chi‐square test, which demonstrated a statistically significant association (*χ*² = 19.4, *df* = 3, *P* < .001). An independent samples *t*‐test was conducted, comparing the number of diagnoses (Counted DX) between Blacks and non‐Blacks. The test revealed no statistically significant difference (*t* = 0.758, *df* = 697, *P* = .449). Additionally, the prevalence of DM was compared between Blacks and non‐Blacks using another chi‐square test. The results showed no significant difference (*χ*² = 0.00649, *df* = 1, *P* = .936, N = 699).

Comparing between teaching versus nonteaching hospitals (urban nonteaching and rural hospitals), there was a difference in the LOS, with average LOS in teaching hospitals significantly longer (5.3 vs 4 days, *P* < .001, *η* ^2^ ≈ 0.0306).

### Gender and Background Diseases

**Figure 2:**
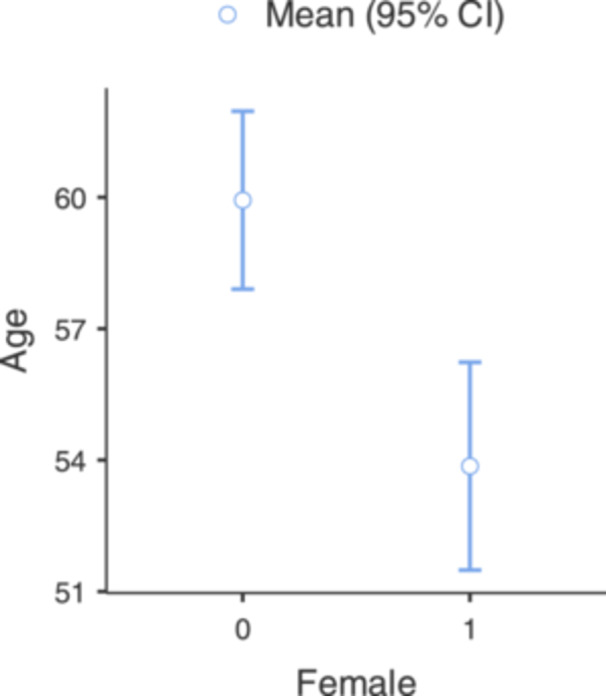
One‐way ANOVA results for age by gender. Differences in the hospitalization characteristics between genders, with women found to be hospitalized at a younger age than men. ANOVA, analysis of variance; CI, confidence interval.

Additional differences in the hospitalization characteristics were found between genders, with women found to be hospitalized at a younger age than men (54 vs 60 years, *P *< .001, *η* ^2^ ≈ 0.0214, Figure 2), while men stay longer in the hospitals than women (5.3 vs 4.6 days, *P* = .033, *η* ^2^ ≈ 0.0064). Analyzing background diseases, we found no statistical evidence between women and diabetic mellitus (Pearson's *r* = −0.019, *df* = 714, *P* = .58), and between women and HIV (Pearson's *r* = −0.020, *df* = 714, *P* = .58). A slight negative correlation was found between women and hematopoietic malignancy (Pearson's *r* = −0.078, *df* = 714, *P* = .037).

Finally, patients with NOE without a background of DM were found to be hospitalized at a significantly younger age than patients with DM (47.5 vs 64 years, *P* < .001, *η* ^2^ ≈ 0.1853, Figure 3). No in‐hospital deaths occurred in this cohort; accordingly, mortality was not analysed.

**Figure 3:**
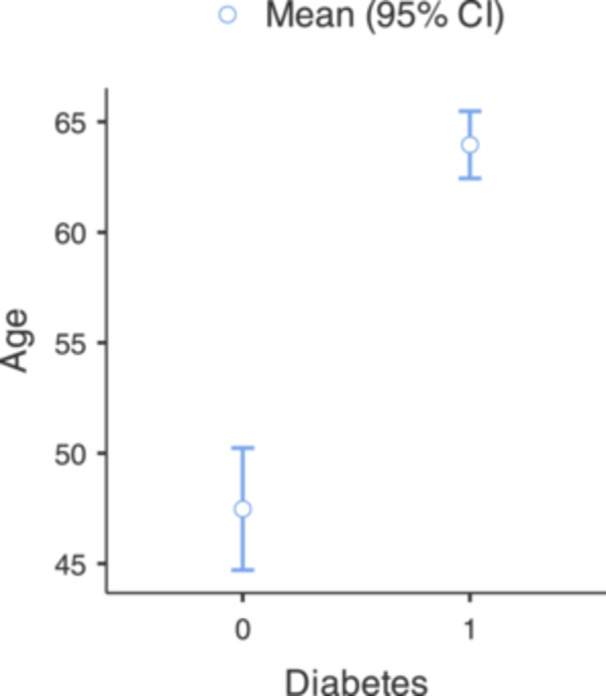
One‐way ANOVA results for age by diabetic status. Patients with NOE without diabetes were hospitalized at significantly younger ages compared to those with diabetes. ANOVA, analysis of variance; NOE, necrotizing otitis externa.

## Discussion

Given the infrequency of incidence of NOE, few studies dealt with demographic characteristics.^1^, ^10^ Recent studies mainly focused on the different outcomes in aged groups^10^ and assessment of management and treatment.^1^ The current study utilized a large sample of patients from the NIS database to conduct an analysis of the characteristics, demographics, and outcomes of hospitalized patients with NOE.

In the first published description of the disease, it was described as prevalent predominantly in elderly and male population. Sylvester et al^10^ found that elderly (age above 65 years) patients are prone for longer hospital stay. In line with those findings, the current study found age to be the most significant predictor of hospital LOS (Figure 1).

Black patients were found to have longer duration of hospitalization compared to white patients. In addition, our analysis suggests that while Blacks tend to have lower income levels based on their ZIP codes, there are no significant differences in the prevalence of DM or the number of background diseases compared to non‐Blacks. Hatch et al^11^ reported that no statistically significant differences in the mean LOS based on race, but they did find race as a significant factor in patient outcomes, particularly for black race. Moreover, people of black race have greater propensity towards DM, and a higher rate of complication due to poor glycemic control and lower healthcare quality in the United States.^12^ Lee et al^13^ found that responsiveness to antidiabetic therapy was closely correlated with the course of NOE.

Recently, many studies have examined the role and outcome of medical teaching‐ compared to a nonteaching hospitals.^14^, ^15^ Teaching hospitals hold a pivotal role by providing training to healthcare professionals, leading the way in the development of innovative surgical techniques and advanced medical treatments, and serving as specialized centers for complex medical cases. The current study found that the length of hospital stay was longer in teaching hospitals, which may reflect differences in patient complexity, referral patterns, or institutional practice characteristics, rather than a direct effect of hospital type.

Additionally, the current study found that women were prone to be hospitalized due to NOE at younger age. First, described by Chandler, the disease mainly afflicts elderly, diabetic males. Similarly, Sylvester et al^10^ found that elderly with NOE were mostly males. Hatch et al^11^ mentioned that males tend to have higher NOE rates than women and are also hospitalized for longer duration. We tried to understand why women are hospitalized at a younger age by looking at different factors. We examined background diseases that are associated with NOE, such as DM, HIV, and hematopoietic malignancy but failed to find statistical evidence. Thus, the observed age difference should be interpreted cautiously and may reflect unmeasured demographic or clinical factors. Further investigation is needed. Also, review of the literature did not find any recent study mentioning female with NOE characteristics, but extensive research in other diseases suggests that gender disparities are important for prevention, diagnostic, treatment, and management. For example, Gao et al^16^ showed that women have higher mortality and worse prognostic after cardio‐vascular event. Furthermore, Sower et al showed that DM obviate the protective effect of sex hormones and increased mortality in women with chronic heart disease with DM compared with women without DM.^17^ This might suggest sex‐related differences in disease behavior warrant further exploration, including in NOE. Healthcare providers may benefit from an understanding that females also experience NOE, potentially at an earlier stage than their male counterparts.

The other group of NOE patients found to be prone to be hospitalized at a younger age were non‐diabetic patients, Sylvester et al^10^ found that pediatric patients with NOE commonly secondary to DM and primary immunodeficiency. Examining a range of variables regarding mortality rates yielded no statistically significant correlation, an outcome that aligns with expectations, given the remarkably low incidence of mortality in question.

Our study has several limitations. While the use of the NIS database provides a large and comprehensive dataset, there are several limitations to consider.

First, the NIS relies on ICD‐10 codes for diagnosis and procedure identification. These codes, while standardized, are subject to inaccuracies in coding and data entry, which can lead to misclassification or incomplete capture of certain conditions. Coding practices may vary between hospitals and regions, potentially introducing variability in the dataset.

Second, the NIS dataset is based on administrative data, which primarily focuses on billing and insurance purposes rather than clinical details. As a result, it may lack important clinical variables such as disease severity, laboratory values, and detailed patient histories, which can be crucial for in‐depth analysis and understanding of clinical outcomes.

Additionally, although several differences in LOS reached statistical significance, the absolute magnitude of these differences was less than 1 day and may therefore have limited clinical relevance. To improve interpretability, we also report median LOS values, which similarly demonstrated small differences across groups.

Lastly, the cross‐sectional nature of the NIS data limits our ability to establish causality or temporal relationships between variables. The data represents a snapshot in time, preventing longitudinal analysis of patient outcomes or disease progression.

Several strengths are associated with data from the NIS. Participation in the NIS is mandatory, and the sampling is weighted to accurately reflect national averages. Moreover, as the largest all‐payer inpatient care database in the United States, the NIS's extensive patient volumes, robust clinical endpoints, and inclusion of real‐world community data help mitigate some of its limitations. These strengths enhance the applicability of our findings across hospitals nationwide, providing valuable insights into healthcare utilization and outcomes in diverse settings.

## Conclusions

The current study utilized the NIS database to examine in‐patients in the United States from 2017 to 2019 with a primary diagnosis of NEO, conducting a comprehensive analysis of cases encompassing characteristics, demographics, and outcomes of hospitalized patients with this condition. Elderly, males, and blacks were associated with longer duration of hospitalization. These associations may relate to factors not captured in the NIS dataset, including clinical severity or social determinants of health, and should therefore be interpreted cautiously. Additionally, patients hospitalized in teaching centers also seem to also have a longer length‐of‐stay. Women's younger age at hospitalization might suggest a different pathophysiology that should be further investigated. Patients without DM are also hospitalized at a much younger age. Clinicians and Healthcare providers should be attentive to variations in disease presentation and behavior within these groups, including the potential occurrence of NOE at an earlier age. Further studies might find the underlying causes for all the above.

## Author Contributions

**Noa Berick**, MD, conceptualization, data analysis, manuscript writing; **Golda Grinblat**, MD, manuscript revision; **Majd Hajouj**, MD, contributing author; **Itzhak Braverman**, MD, manuscript revision, mentoring; **Isaac Shochat**, MD, data analysis, manuscript revision, senior mentorship.

## Disclosures

### Competing interests

None.

### Funding source

None.
